# Influence of proprioceptive training based on ankle-foot robot on improving lower limbs function in patients after a stroke

**DOI:** 10.3389/fnbot.2022.969671

**Published:** 2022-10-26

**Authors:** Yajun Mao, Zhenzhen Gao, Hang Yang, Caiping Song

**Affiliations:** Department of Rehabilitation, The First Affiliated Hospital of Zhejiang Chinese Medical University (Zhejiang Provincial Hospital of Traditional Chinese Medicine), Hangzhou, China

**Keywords:** proprioception, proprioceptive strength training, stroke, lower limbs function, motor function

## Abstract

**Background:**

Proprioception is important for our everyday activity, as it indicates the position, movement, and force on the body. This is important not only for ambulation but also for patients who are diagnosed with stroke.

**Objective:**

This study aimed to evaluate the influence of proprioceptive training on lower limb function in patients after a stroke using an ankle-foot robot.

**Method:**

In total, 60 adult participants who met the criteria were randomly divided into a control group and an experimental group. The control group (RG) was given regular physical activity, and the sensory training group (SG) was given proprioceptive training based on an ankle-foot robot, the rest being the same as RG. Measurements for 10-meter walking time (10MWT), the Berg Balance Scale (BBS), the Fugl-Meyer assessment of lower extremity (FMA-LE), and active range of motion (AROM), passive range of motion (PROM), and ankle joint sensitivity before and after 6 weeks of treatment (30 sessions; five times per week) were assessed.

**Results:**

There was a significant decrease in both 10MWT and ankle joint sensitivity in both groups (*p* < 0.05), while there was a significant increase in BBS, FMA-LE, AROM, and PROM in both groups (*p* < 0.05). A significant relationship was identified between the two groups, the SG group had greater degrees of improvement compared to the RG group.

**Conclusion:**

The proprioceptive training based on an ankle-foot robot could improve proprioception and effectively improve the motor function and walking ability in patients after a stroke. Proprioceptive strength training is recommended to be emphasized in the regular rehabilitation of patients after a stroke.

## Introduction

Proprioception refers to the perception of position, movement, and force on the body (Sherrington, 1907; Proske and Gandevia, 2012). The incidence of proprioceptive deficits after a stroke is 50–65%, which is associated with prolonged hospitalization and decreased motor and functional recovery (Carey, 1995; Connell et al., 2008; Tyson et al., 2008; Morris et al., 2013; Semrau et al., 2013). Proprioception deficits after a stroke can cause difficulty in mobility and loss of confidence in patients, with a negative long-term impact on simple daily activities. For example, the decrease in proprioception entails these patients to look down to observe the position of their feet while walking. In addition to this, they cannot reach, grasp, and manipulate objects at will. A secondary complication, such as hemineglect, refers to the refusal of using their affected limb possibility due to uncertainty, fear, insecurity, and a lack of confidence. Due to this long-term hemineglect disorder, motor function further deteriorates, greatly limiting the improvement of quality of life (Carey et al., 2018). In support, for patients to complete an active movement, a series of processes are required. These processes include an intact relationship between both the sensory system and active movement. Thus, the central nervous system has to commence with motor preparation, action execution, and overall monitoring function. While the motor system is required for the preparation and execution of function (Perruchoud et al., 2014), all these systems are required for any active movement to be executed. In advanced motor behaviors, the brain must integrate sensory inputs to accurately assess the surrounding environment and generate corresponding motor outputs. Motor adaptation refers to the ability to continuously adjust motor strategies to adapt to changes in the environment, which is based on feedback from sensory input (Papale and Hooks, 2018). Sensory signals affect motor function by feeding external environmental information and internal physiological state to guide the activation of the motor system. Problems encountered in any aspect of the process will cause motor dysfunction.

A study has shown that the improvement of proprioception is closely correlated with advanced motor function (Grant et al., 2018). Proprioceptive training is a form of somatosensory intervention aimed at enhancing proprioceptive function. The previous study showed that sensory stimuli, such as repetitive skin stimulation (tactile simulation) (Timm and Kuehn, 2020), passive limb movement training (passive range of motion, PROM) (Dechaumont-Palacin et al., 2008), repetitive sensory discrimination exercises and active sensory, and motor feedback training, contribute to proprioceptive function and motor function after a stroke (Rowe et al., 2017; Ingemanson et al., 2019; Vahdat et al., 2019).

The sensory sensitivity of the lower extremity, especially the ankle joint, which is the basis of the function of the entire lower extremity (LE), plays a key role in the balance and motor function of the lower extremity. Damage to ankle motor control has been identified as an important cause of walking inability after a stroke. Conventional rehabilitation often emphasizes neuromuscular facilitation to improve dorsiflexion or includes the use of ankle-foot orthoses (AFO) to reduce ankle plantarflexion and achieve walking. Sensory training, especially proprioceptive training, is often ignored. Proprioception awareness is the most important factor in predicting the ability to balance among strength, range of motion, and proprioception of the ankle joint in patients with stroke (Cho and Kim, 2021).

This study included hemiplegic patients with sensory dysfunction at 1–6 months after stroke. The experimental group was given repetitive and strengthened proprioceptive training based on the ankle-foot robot, as well as regular training. This enables further observation of the effect of the motor function of the lower limbs influence in patients after a stroke.

## Materials and methods

### Study design

This study is a randomized controlled trial (RCT). The pre-and post-experimental designs were carried out from 2021 to 2022 at the Department of Rehabilitation, the First Affiliated Hospital of Chinese Medical University. This study is approved by the Ethics Committee of the First Affiliated Hospital of Zhejiang Chinese Medical University (No: 2022-K-269-01).

### Participants

This study included 60 patients with proprioceptive impairment after a stroke following the eligibility criteria.

Inclusion criteria: The patients (1) should be 18–75 years old, (2) should have been diagnosed with their first stroke, confirmed by magnetic resonance imaging (MRI) or computed tomography scan (CT scan), (3) should have been diagnosed of stroke in the past 6 months, (4) should possess unilateral limb sensory and motor dysfunction (5) should have a National Institutes of Health Stroke Scale (NIHSS)score of ≤ 15, and (6) should walk independently for ≥ 10 meters (walk ability).

The patients were excluded based on the following criteria: (1) any muscle tone tension of the LE increases to the extent that modified Ashworth grade is ≥ 2, (2) any joint contracture of the lower limbs, (3) a history of abnormal gait in the past, (4) patients with other medical conditions that restrict rehabilitation, and (5) no previous proprioceptive disorders.

### Randomization

**Figure 1:**
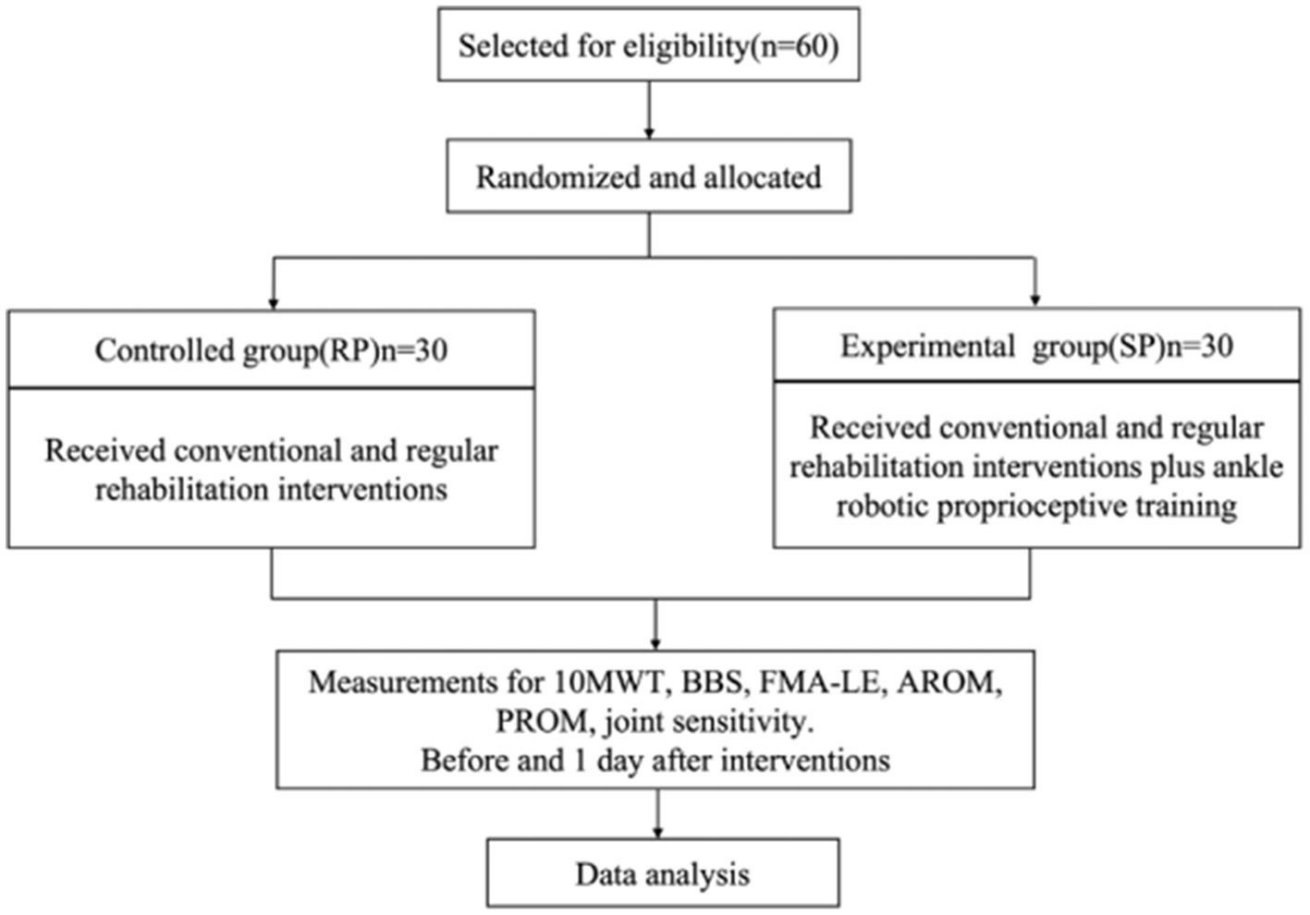
Flowchart of the participants.

All the participants were randomly divided into the control group (RG) and the experimental group according to the random number table created by the computer. Each group has 30 patients (Figure 1).

### Ethical approval

This study was approved by the Ethics Committee of the First Affiliated Hospital of Zhejiang Chinese Medical University (No: 2022-K-269-01). Participants were informed regarding the procedure of this study, and participation was completely voluntary and they may stop participating at any time without any negative consequences. All patients and (or) their legal guardians in the study voluntarily agreed to participate and sign the informed consent before the start of the trial.

### Interventions

Conventional exercise therapy was performed including joint stretching, muscle strengthening, and endurance training. This group received the same treatment as RG, and an additional robot-guided ankle proprioceptive training combined with thermal-tactile stimulation.

The treatment duration lasted for 6 weeks (one time a day, 5 days per week, a total of 30 sessions).

**Figure 2:**
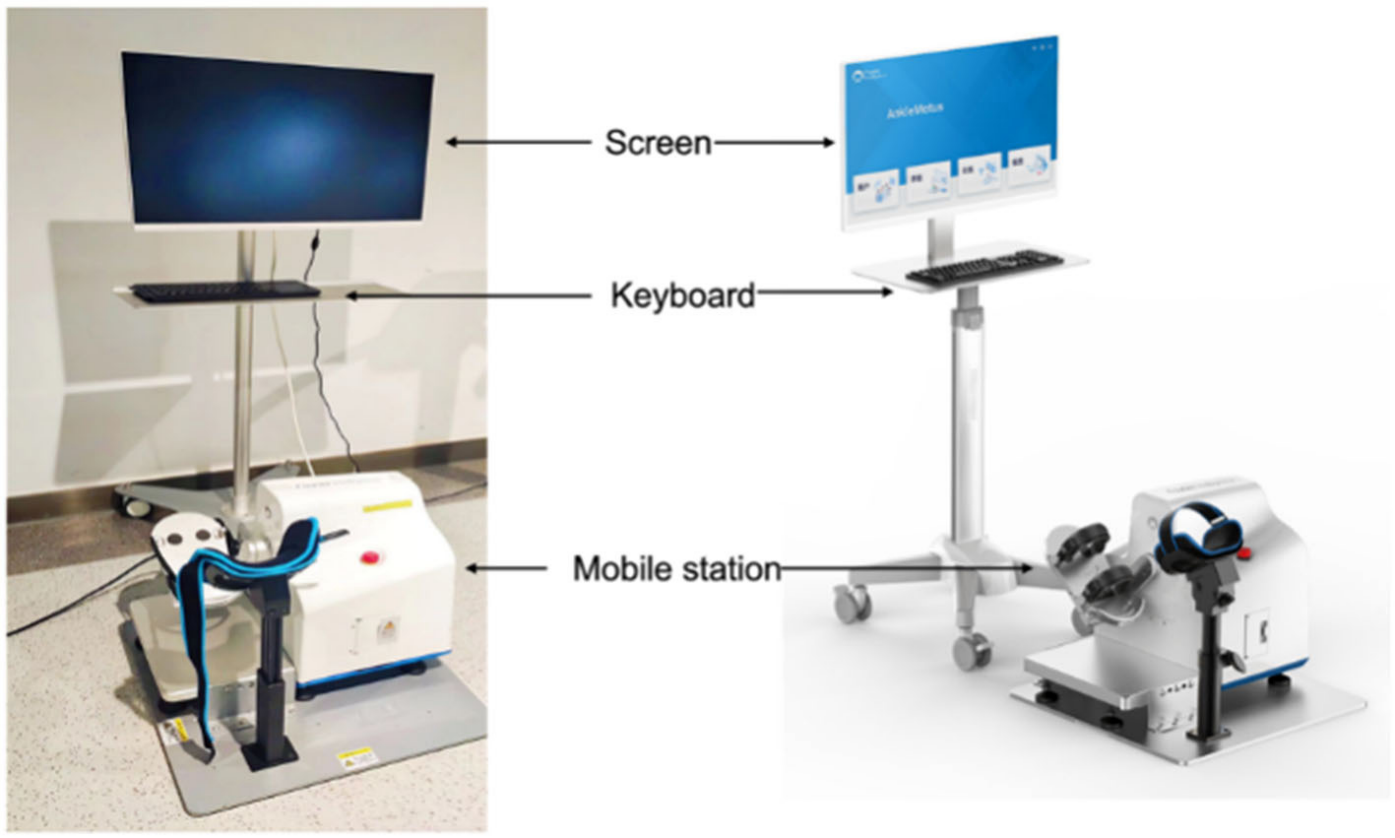
AnkleMotus M1A.

Proprioceptive training: Ankle sensory training with robot guidance: Assessment and training were performed using AnkleMotus M1A produced by Shanghai Fourier Intelligent Technology Co., Ltd. (see Figure 2).

**Figure 3:**
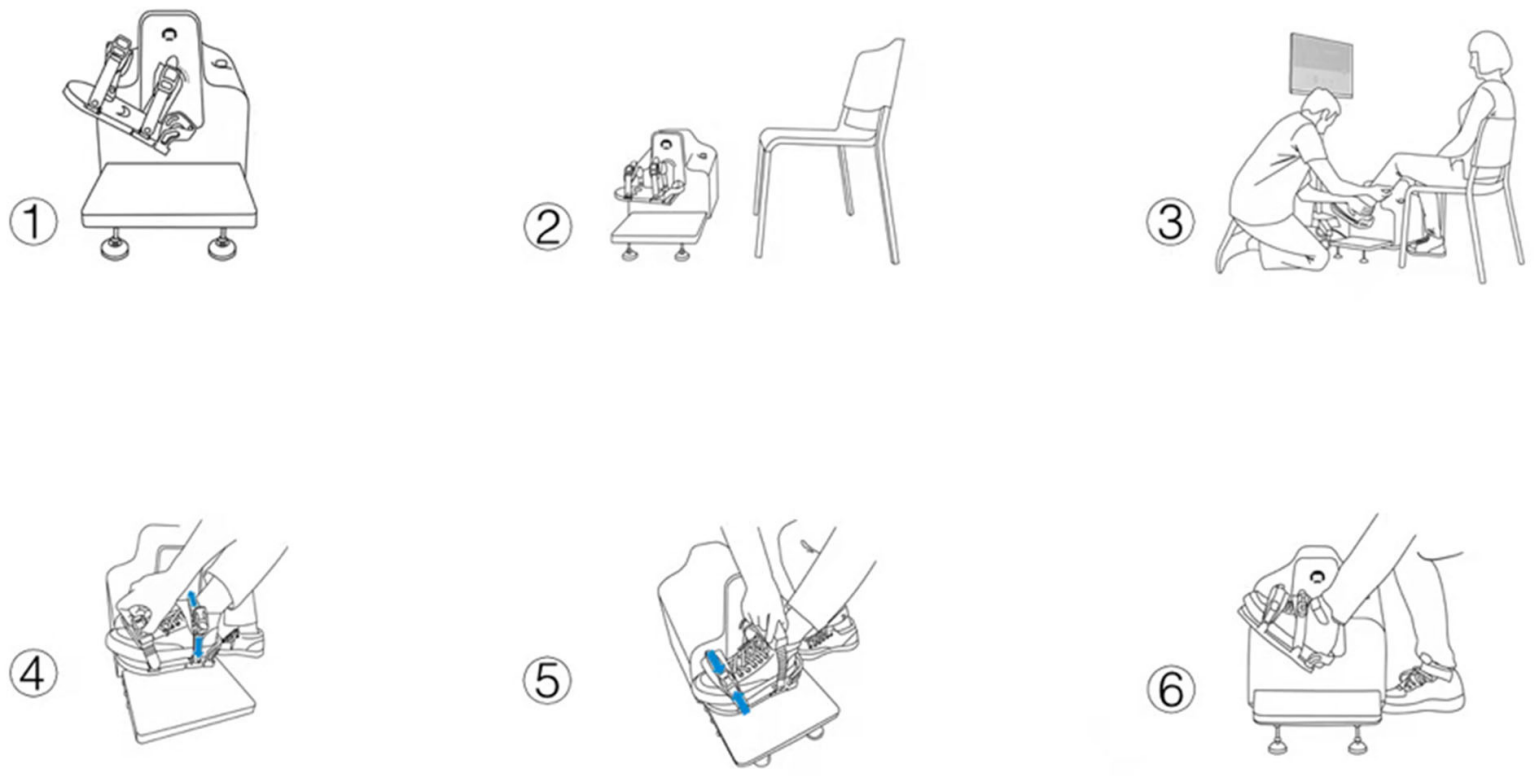
The procedures for the training position.

Training position: The participants were asked to sit comfortably on a liftable chair that is about 20–30 m away from the machine. The affected foot was strapped on the pedal. The starting position is the neutral position of the ankle joint at 0 degree (see Figure 3). Assessment: The evaluation module was selected to measure the PROM, active range of motion (AROM), and ankle joint sensitivity, respectively. Training program: This program contains joint position awareness training and joint kinaesthetic training. Joint position awareness training involved using the ankle-foot robot to perform passive joint stretching with a range of motion of PROM + 3° (maximum range of motion: plantar flexion 40°, dorsiflexion 24°), and then the patient was actively restored to the initial position. Joint kinaesthetic training involved first performing passive training for 10 min, with the ankle joint being in a 0-degree position and passively moving at the speed of one degree per second controlled by the ankle-foot rehabilitation robot. The patient then performed ankle joint training while watching the movement, followed by the same ankle joint training, but this time with their eyes closed while following the therapy instructions. Both ankle joint training was performed for 5 min. Next, the patient performed active training for 10 min. The procedure was as follows: The patient moved the ankle joint for the first 10 min according to the requirements of the game displayed on the screen, with their eyes looking at the screen; then, the patient was made to look at the ankle joint and actively move the ankle joint for the next 10 min as instructed by the therapist according to the game requirements. Whenever the activity was performed correctly, the system outputs a piece of reward music, and the therapist gave the same encouragement (auditory feedback) at the same time. Different active training modules were chosen (assistance exercise or resistance exercise) according to the muscle strength of the patient.

### Assessment

#### Time

Both groups were tested before the start of the treatment and 1 day after the end of treatment (pre and post-test). The primary outcomes of this study included the 10-meter walking time (10MWT) and Berg Balance Scale (BBS) to assess walking and balance ability, while the secondary outcomes included Fugl-Meyer assessment of lower extremity (FMA-LE), AROM, PROM, and ankle joint sensitivity corresponding to the ankle joint proprioceptive training.

(1) 10MWT. It is a performance measure used to assess walking speed in meters per second over a short distance. It can be employed to determine functional mobility and gait. The total time taken to ambulate 6 meters (m) is recorded to the nearest hundredth of a second. The faster the speed is, the better the walking ability is. A clear pathway of at least 10 m in length was designed in a quiet room. The start and endpoints of a 10-m walkway were measured and marked. A mark was added at 2 and 8 m. All participants started to walk according to the instructions, and the time was recorded as they walked past through the 2 and 8 m marks. We took an average of three walking measurements. The test results indicated the quality of the walk; the lower the time, the better the quality of the walk. Then, the functional mobility, gait, and vestibular function of the patients were evaluated.

(2) BBS. It was used to evaluate the balance function. The result was often used to objectively determine the ability of a patient (or inability) to safely balance during a series of predetermined tasks.

(3) FMA-LE. It was used to evaluate the LE motor function; the more the score, the better the lower limb is. It is a widely used and recommended scale for the evaluation of post-stroke motor impairment.

(4) Biomechanical index. It consists of AROM, PROM, and ankle joint sensitivity. They were evaluated through AnkleMotus M1A. Each test result was performed three times and took an average of three measurements.

The participants sat in a chair with the knee flexed and the affected foot strapped to the pedal. The ankle joint was taken in a neutral position, which was confirmed as 0 degree on the computer.

#### AROM

The patient was asked to actively move the ankle from the neutral position to the maximal dorsiflexion/plantarflexion while receiving visual feedback on the movement and testing the angle on the computer.

#### PROM

The ankle of the patient was passively stretched to the maximum dorsiflexion/plantarflexion position by the machine.

#### Ankle joint sensitivity

The ankle joint of the patient was passively plantarflexed to a certain angle (target angle), the machine was maintained at that ankle position for 10 s so that the patient (with their eyes closed) can remember the angle. Then, the machine moved the ankle joint back to the 0-degree position. Next, the patient was asked to position their ankle to the target angle to feel the muscle contraction. The error angle from the set angle was recorded as joint sensitivity. The difference in degree was used to determine the joint sensitivity of the ankle; thus, the lower the ankle joint sensitivity is, the better the ankle is.

### Statistical processing

Statistical analysis was performed using SPSS version 20.0 software. Measurement data were expressed as mean ± standard deviation (x ± s); the paired *t-test* was used for comparison before and after treatment within a group, and the *t-test* was used for comparison between the two groups. The χ^2^ test was used to analyze the enumeration data. *P* < 0.05 was considered statistically significant.

## Results

### Baseline characteristics

The baseline characteristics including the age, gender, type of stroke, hemiplegia side, medical history time, and the NIHHSS score in both groups were assessed. There were no significant differences in the baseline characteristics between the two groups (*p* ≥ 0.05) (Table 1).

**Table 1:** Baseline characteristics of the patients in two groups.

**Group**	**Age**	**Gender (** ***n*** **)**		**Type of stroke (** ***n*** **)**		**Hemiplegia side (** ***n*** **)**		**NIHSS**	**Medical history**
		**Male**	**Female**	**Intracerebral**	**Cerebral**	**Right**	**Left**		
				**hemorrhage**	**infarction**				
SG group	60.00 ± 9.10	20	10	11	19	12	18	7.75 ± 4.11	97.73 ± 21.23
RG	61.10 ± 7.88	18	12	10	20	10	20	7.77 ± 1.62	88.13 ± 21.55
*t*-value	0.410							−0.339	1.056
χ^2^ value		0.287		0.073		0.287			
*p*-value	0.684	0.592		0.787		0.592		0.736	0.295

### Biomechanical index

There was no significant difference in the AROM, PROM, and joint sensitivity between the two groups before training (*p* > 0.05). The within-group comparisons after training showed a statistical increase in AROM and PROM in both control and experimental groups (*p* < 0.05), while a statistical decrease in joint sensitivity (${\text{t}}_{1,2,3}^{\text{Δ}}$ = −9.855, −22.75, 9.798) (*p* < 0.05), which meant that the biomechanical index improved in both groups. The in-between comparison showed an insignificant improvement in all biomechanical indexes, which is better in the sensory training group (SG) (Table 2).

**Table 2:** Biomechanical index assessments in AROM, PROM, joint sensitivity.

**Group**	**Before training**			**After training**		
	**AROM**	**PROM**	**Joint sensitivity**	**AROM**	**PROM**	**Joint sensitivity**
Sensory training group	20.09 ± 3.27	33.17 ± 2.51	6.28 ± 1.91	27.01 ± 3.31	55.62 ± 3.96	2.76 ± 0.41
Control group	20.10 ± 4.47	33.41 ± 1.98	5.93 ± 1.35	23.43 ± 4.14	42.04 ± 5.06	3.83 ± 0.40
*t*-value	*t*_1_ = 0.987	*t*_2_ = −0.43	*t*_3_ = 0.835	$t_{1}^{*}$= 3.853, $t_{1}^{\text{Δ}}$ = −9.855	$t_{2}^{*}$= 0.225, $t_{2}^{\text{Δ}}$ = −22.75	$t_{3}^{*}$= 10.246, $t_{3}^{\text{Δ}}$ = 9.798
*P*-value	0.328	0.669	0.407	0.000	0.000	0.000
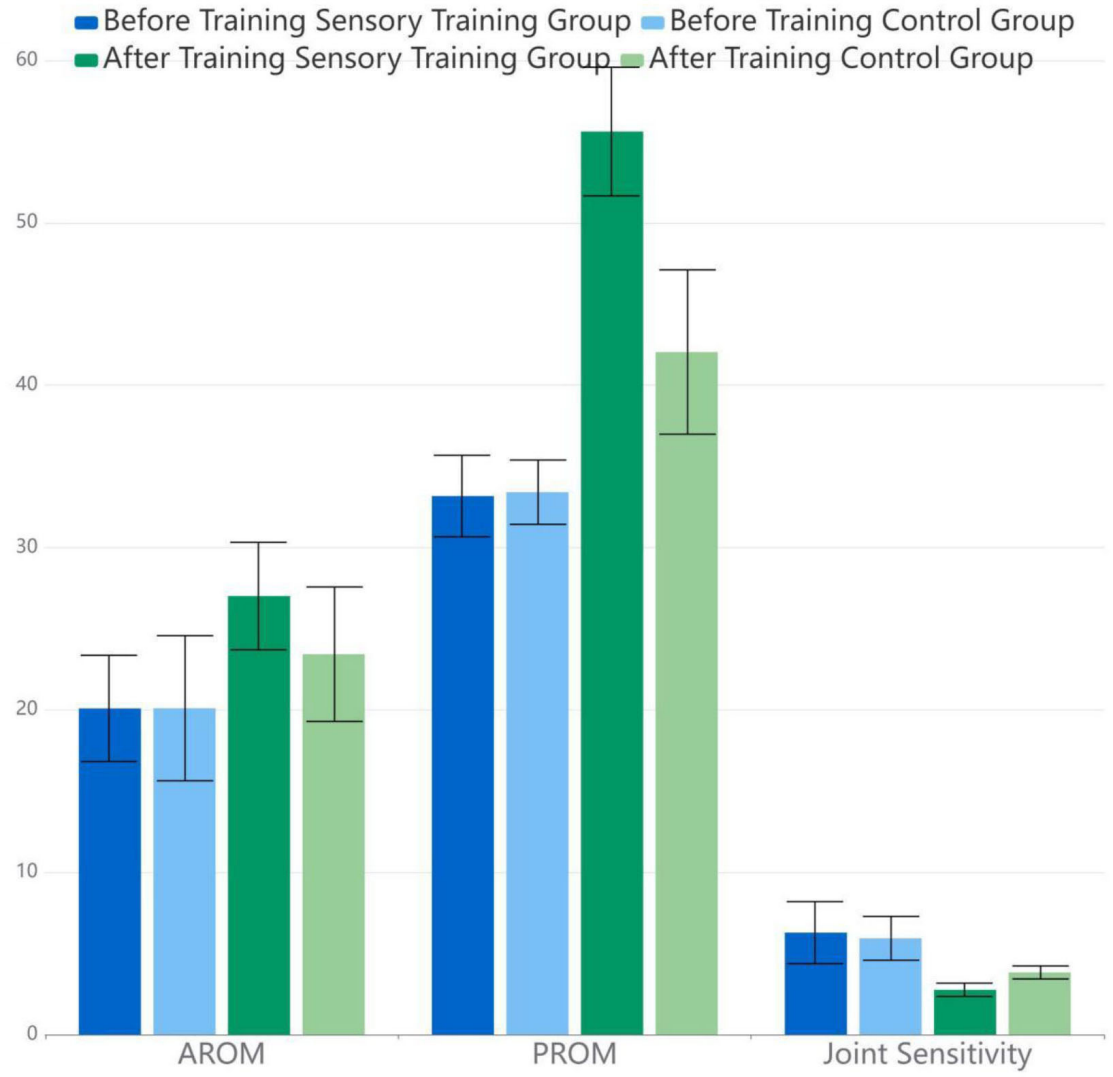						

*t*: the results of AROM, PROM and joint sensitivity between sensory training and control groups before treatment.

t^*^: the results of AROM, PROM and joint sensitivity between sensory training and control groups after treatment.

*t*^Δ^: Comparasting results of AROM, PROM and joint sensitivity before and after treatment in sensory training.

### Motor function

There were no significant differences in the 10MWT, BBS, and FMA-LE between the two groups before training (*p* > 0.05). The within-group comparisons after training showed a statistically significant increase in BBS and FMA-LE in both the RG and the experimental group (*p* < 0.05), while a statistically significant decrease in 10MWT (${\text{t}}_{1,2,3}^{\text{Δ}}$ = −29.359, −63.349, 24.131) (*p* < 0.05) was observed, which meant that the motor function improved in both groups. Moreover, the comparison of the in-between groups showed that 10MWT, BBS, and FMA-LE had improved more in the SG (*p* < 0.05) (Table 3).

**Table 3:** Lower extremity motor function assessment.

**Group**	**Before training**			**After training**		
	**FMA-LE**	**BBS**	**10 MWT(s)**	**FMA-LE**	**BBS**	**10 MWT(s)**
Sensory training group	18.37 ± 1.75	19.97 ± 3.13	95.97 ± 14.92	24.57 ± 1.63	31.87 ± 2.70	71.93 ± 12.73
Control group	17.09 ± 1.74	19.80 ± 2.91	97.33 ± 15.17	21.20 ± 1.79	26.97 ± 2.74	82.07 ± 13.84
*t*-value	*t*_1_ = 1.033	*t*_2_ = 0.213	*t*_3_ = −0.352	$t_{1}^{*}$= 7.612, $t_{1}^{\text{Δ}}$ = −29.359	$t_{2}^{*}$= 6.983, $t_{2}^{\text{Δ}}$ = −63.349	$t_{3}^{*}$= 2.951, $t_{3}^{\text{Δ}}$ = 24.131
*p*-value	0.306	0.832	0.726	0.000	0.000	0.005
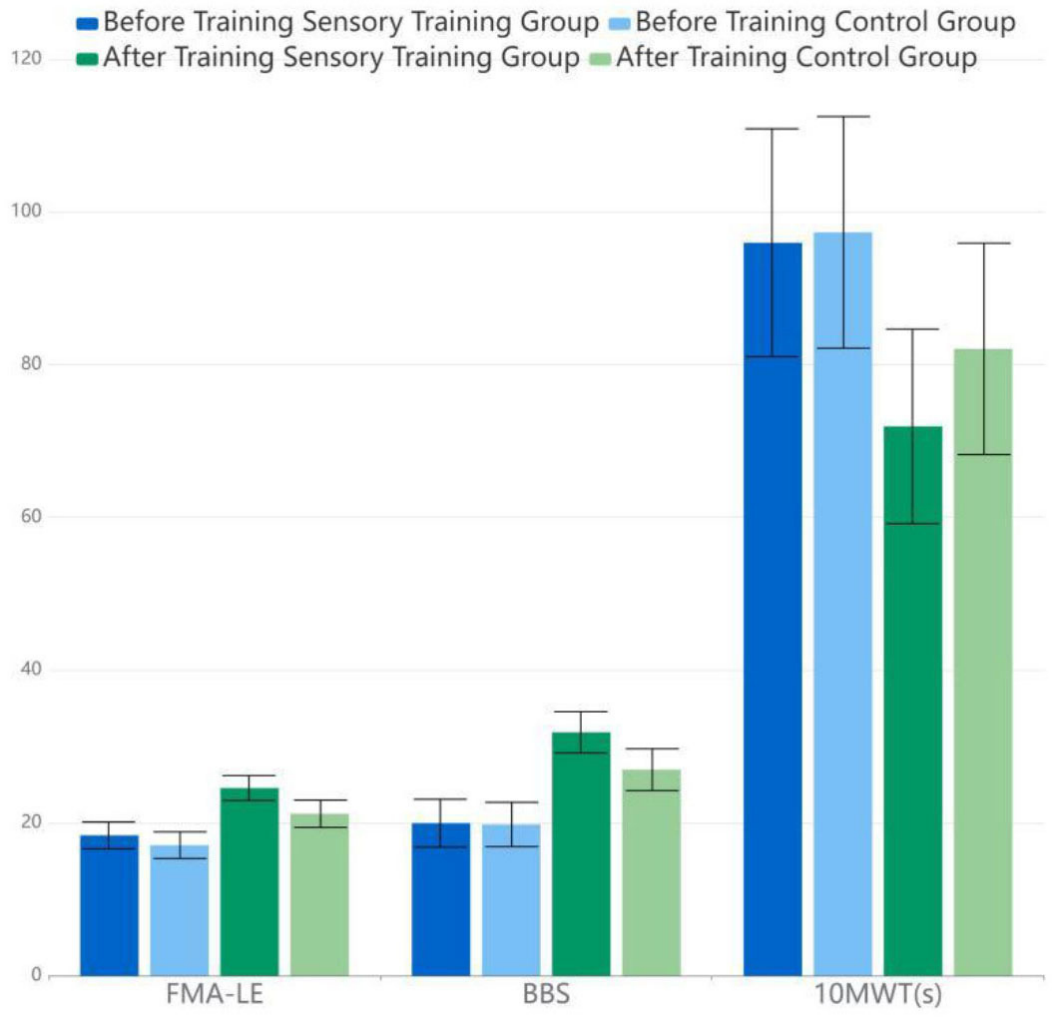						

*t*: the results of FMA-LE, BBS, 10MWT between sensory training and control groups before treatment.

t^*^: the results of FMA-LE, BBS, 10MWT between sensory training and control groups after treatment.

*t*^Δ^: Comparasting results of FMA-LE, BBS, 10MWT before and after treatment in sensory training.

## Discussion

Common commercial, industrial serial robots with redundant manipulators have been successfully adopted and further developed in precise automation processes for a variety of applications. The remote center of motion constraint has been established successfully. The corresponding end-effector tracking errors asymptotically converge to zero. The experiments were conducted in a laboratory setup environment using KUKA LWR4+ to validate the effectiveness of the proposed control strategy (Su et al., 2020). A hybrid shared control approach based on EMG and artificial potential field was exploited to avoid obstacles, according to the repulsive force and attractive force and to enhance the human perception of the remote environment based on force feedback of the mobile platform (Luo et al., 2020). Proprioception is a special sense that includes joint motion and position sense, mainly including the static awareness of joint position and the kinaesthesia of joint motion (joint motion or acceleration perception). Sensory dysfunction after a stroke is associated with slower recovery, decreased motor function, and poorer rehabilitation effects. We enrolled and divided the 60 participants who met the criteria. Fortunately, all participants were able to complete this study. We observed the influence of proprioception training in lower limbs based on ankle-robot.

The within-group comparison conducted post-test in both groups revealed a statistically significant improvement in the walking speed, balance ability, and biomechanical index (*p* < 0.05 in all measures). These results further confirmed that there were significant differences between individuals in mobility, balance, balance confidence, and independence of daily living with and without mild proprioceptive deficits (Rand, 2018).

As a result, the in-between group comparison showed statistically better results in the SG in the 10MWT, BBS, and biomechanical index in AROM, PROM, and ankle joint sensitivity than that in the RG (*p* < 0.05) in all measures. The two groups trained their motor control by repeating the training multiple times, of which the theory is based on motor re-learning. Moreover, the SG strengthened proprioceptive facilitation, with audio and visual feedback in the process, and achieved better results in the assessments of biomechanics and LE motor function. This seems to be related to the sensory training equipment, ankle-foot-robot, which could accurately evaluate the AROM, PROM, and ankle joint sensitivity, precisely locate the training angle, and provide feedback stimulation in time. Systematic review studies have also shown that robot-assisted therapy can significantly improve the balance function in patients after a stroke (Zheng et al., 2019). Proprioceptive training can effectively improve the balance ability, gait speed, trunk control, and basic functional activities of patients with stroke (Apriliyasari et al., 2022). Vahdat et al. (2019) reported that it could improve motor accuracy in patients with chronic stroke by using a single-shot robotic-controlled proprioceptive training with feedback, and also it could induce functional connectivity changes in sensorimotor networks, which are consistent with our findings.

Ankle biofeedback training can significantly improve ankle muscle strength, balance, and gait in patients with stroke (Kim et al., 2016). The pain, sensory impairment, and muscle weakness of the affected foot and ankle are responsible for balance, walking ability, and the fear of falling in patients after a stroke (Gorst et al., 2016). Regarding the 10MWT, the overall walking speed was improved compared with that before the training, which could indicate that the confidence level of the patients in walking safely was improved. From the results of BBS, the score increased significantly in the SG. However, the tool of BBS is relatively subjective as there may be some intervention bias caused by a person. BBS could not evaluate ankle stability, especially the static and dynamic balance of the ankle.

In addition, different from the conventional ankle joint passive and neurodevelopmental training, this ankle-foot robot greatly improves the fun and efficiency of the rehabilitation process, combining the advantages of task-oriented training in the form of a game and rewarding with music biofeedback. It was reported that visual feedback has a positive effect on balance when patients with stroke perform ankle strategy exercises (Jeon and Choi, 2015). For patients with proprioceptive deficits, methods including visual compensation are usually used to improve their motor and daily life. However, some research studies against the traditional views have shown that visual compensation does not help many patients with stroke compensate for the impaired sense of position (Herter et al., 2019) and motor performance (Semrau et al., 2018). An effective way to improve impaired proprioception is through intensive training, not by simply compensating. Our research emphasizing intensive proprioceptive training is also based on the theory of motor re-learning and repeated stimulation.

Regarding the biomechanical index, the measurements of AROM and PROM were statistically increased in both groups after training; thus, the results of the SG improved statistically (*p* < 0.05). We also measured the ankle joint sensitivity with the ankle-foot robot, which also demonstrated more statistical sensitivity in the SG (*p* < 0.05). Previous research reported that ankle proprioceptive training often leads to a more upright trunk position and better posture control (Ahmad et al., 2020). The measurement of proprioception is a little complicated. In this study, we took the error angle deviating from the set angle as the joint sensitivity value by using the ankle-foot robot. This measurement method can initially reflect the proprioceptive situation of the ankle joint.

As it was reported, the walking ability, balance function, and biomechanical indexes in the SG were significantly improved. It is more likely to describe that the robot-based ankle joint sensory training increased the sensory sensitivity input by expanding the angle of the ankle movement and strengthening the interaction of the ankle-foot. The control of postural stability, the balance stability of standing and walking, and the enhancement of sensory input further promote the establishment of walking safely, so the walking speed is improved.

From the results, it was discovered that a blind spot of traditional rehabilitation training was present as it ignores or weakens proprioceptive reinforcement. The present study showed that there is an anatomical basis for improving motor function through sensory training. First, in the basal ganglia circuit, the sensory afferents directly or indirectly project to the brainstem, the cerebellum, the subcortex, and the cortex. The basal ganglia are connected to the frontal lobe, the limbic system, and the sensory system through the neural circuit. This circuit is involved in motor control and the integration of cognitive, emotional, and sensorimotor information. Furthermore, the basal ganglia circuit can be modulated by specialized dopamine receptors. Second, in the cerebellar circuit, the cerebellum directly receives abundant sensory afferent fibers, which play an important role in the movement and regulation of motor coordination (Draganova et al., 2021). The cerebral cortex—cerebellar circuit connects the frontal lobes, the pons, the cerebellar cortex, the deep cerebellar nucleus, the ventrolateral thalamic nucleus, and the motor cortex, providing an anatomical basis for the regulation of motor coordination. Furthermore, there are dual fiber connections between the basal ganglia, the sensorimotor cortex, and the cerebellum through virus tracing techniques (Welniarz et al., 2021). The cerebral-basal ganglia-cerebellum circuit plays an important role in motor, cognitive, emotional, and sensory functions in patients with movement disorders. There are few previous clinical trials on strengthening sensory training, especially proprioception, to promote the recovery of motor function. Some systematic reviews have found insufficient evidence to support interventions like sensory training for post-stroke rehabilitation but further research is still needed.

There are some limitations to the study. First is the limitation of the small sample size that may have an impact on the accuracy of the test data since the data collected is just limited to one center. In terms of intervention time, the second limitation is that a total of 30 sessions conducted in 6 weeks may not improve the prognosis. Finally, there is a lack of follow-up. We hope to optimize the standardized rehabilitation training program through further long-term follow-up of data.

When assessing the ability to balance, BBS is more subjective. Taking that into consideration, we will attempt to use gait analysis equipment so that it is more objective to make assessments from multiple dimensions. From the analysis of the results, we have not further studied whether changes in joint sensitivity are related to changes in AROM and PROM and whether there is a certain correlation between balance and walking speed.

## Conclusion

The novelty of this study is that we first proved the effect of proprioceptive training in a series of patients using an ankle-foot robot. Proprioceptive strength training is worth to be further applied in patients after a stroke. In addition to routine exercise training, repetitive and intensive proprioceptive training based on ankle-foot robots is conducted for 1–6 months in stroke and hemiplegia patients with sensory and motor dysfunction, which can effectively improve the motor functioning and walking ability of the patients. In particular, it improves PROM, AROM, and joint sensitivity and also improves the motor function, balance function, and walking ability of patients. The robot-based ankle joint sensory training increased the sensory sensitivity input by expanding the angle of ankle movement and strengthening the interaction of the ankle-foot. The control of postural stability, the balance stability of standing and walking, and the enhancement of sensory input further promote the establishment of walking safety, so the walking speed is improved. Regarding the 10MWT, the overall walking speed was improved compared with that before the training, which could indicate that the confidence level of the patients in walking safety was improved. This study suggests that patients with stroke should undergo proprioceptive training while performing routine exercise rehabilitation therapy.

## Data availability statement

The original contributions presented in the study are included in the article/supplementary material, further inquiries can be directed to the corresponding author/s.

## Ethics statement

The studies involving human participants were reviewed and approved by the Ethics Committee of the First Affiliated Hospital of Zhejiang Chinese Medical University (No: 2022-K-269-01). Written informed consent to participate in this study was provided by the patient/participants.

## Author contributions

CS: guarantor of integrity of the entire study, definition of intellectual content, statistical analysis, and manuscript review. YM: study concepts and study design. YM and HY: literature research and data analysis. ZG: clinical studies. YM and ZG: data acquisition. YM, HY, and ZG: manuscript editing. All authors contributed to the article and approved the submitted version.

## Funding

This work was supported by the general research program of the Zhejiang Provincial Department of Health (Grant No. 2022KY922). It was also partially supported by the TCM Rehabilitation Service Capacity Improvement Project (inheritance and development of TCM).

## Conflict of interest

The authors declare that the research was conducted in the absence of any commercial or financial relationships that could be construed as a potential conflict of interest.

## Publisher's note

All claims expressed in this article are solely those of the authors and do not necessarily represent those of their affiliated organizations, or those of the publisher, the editors and the reviewers. Any product that may be evaluated in this article, or claim that may be made by its manufacturer, is not guaranteed or endorsed by the publisher.
